# Development and validation of a clinical-radiomics nomogram for the early prediction of Klebsiella pneumoniae liver abscess

**DOI:** 10.1080/07853890.2024.2413923

**Published:** 2024-10-11

**Authors:** Li Gu, Tao Ai, Qing Ye, Yihang Wang, Han Wang, Dong Xu

**Affiliations:** a Department of Infectious Diseases, Tongji Hospital, Tongji Medical College, Huazhong University of Science and Technology, Wuhan, Hubei, China; b Department of Radiology, Tongji Hospital, Tongji Medical College, Huazhong University of Science and Technology, Wuhan, Hubei, China; c Tongji Hospital, Tongji Medical College, Huazhong University of Science and Technology, Wuhan, Hubei, China; d School of Artificial Intelligence, Beijing University of Posts and Telecommunications, Beijing, China; e Department of Gastroenterology, Tongji Hospital, Tongji Medical College, Huazhong University of Science and Technology, Wuhan, Hubei, China

**Keywords:** Klebsiella pneumoniae, pyogenic liver abscess, radiomics, nomogram, computed tomography, diagnosis, prediction

## Abstract

**Background and aim:**

Pyogenic liver abscess (PLA) is a devastating and potentially life-threatening disease globally, with Klebsiella pneumoniae liver abscess (KPLA) being the most prevalent in Asia. This study aims to develop an effective and comprehensive nomogram combining clinical and radiomics features for early prediction of KPLA.

**Methods:**

255 patients with PLA from 2013 to 2023 were enrolled and randomly divided into the training and validation cohorts at a 7:3 ratio. The differences between the two cohorts of patients were assessed *via* univariate analysis. The radiomics features were extracted from imaging data from enhanced CT of liver abscesses. The optimal radiomics features were filtered using the independent sample t-test and least absolute shrinkage and selection operator, and a radiomics score (Rad-score) was calculated by weighting their respective coefficients. Clinically independent predictors were identified from the clinical data and combined with the Rad-score to develop a nomogram by multivariate logistic regression. The predictive performance was evaluated using the area under the receiver operating characteristic curve (AUC), calibration curve, and clinical decision curve.

**Results:**

The nomogram incorporated four clinical features of diabetes mellitus, cryptogenic liver abscess, C-reactive protein level, and splenomegaly, and the Rad-score that was constructed based on seven optimal radiomics features. It had an AUC of 0.929 (95% CI, 0.894-0.964) and 0.923 (95% CI, 0.864-0.981) in the training and validation cohorts, respectively. The calibration and decision curves showed that the nomogram had good agreement and clinical applicability.

**Conclusions:**

The clinical-radiomics nomogram performed well in predicting KPLA, hopefully serving as a reference for early diagnosis of KPLA.

## Introduction

Pyogenic liver abscess (PLA) is an infected, space-occupying lesion of the liver caused by a variety of bacteria, which can result in life-threatening complications such as extrahepatic metastatic infections, sepsis, multiple organ failure, and shock. Before the 1980s, Escherichia coli was the most common pathogen of PLA. However, over the past three decades, Klebsiella pneumoniae (KP) has gradually replaced Escherichia coli as a major causative organism, particularly in Asia [1, 2]. Based on virulence, KP is classified into two types: classic Klebsiella pneumoniae (cKP), which primarily affects immunocompromised patients, and hypervirulent Klebsiella pneumoniae (hvKP), which, however, can infect young, healthy, and immunocompetent individuals and cause fatal community-acquired infections [3]. Previous studies have shown that hvKP was frequently linked to Klebsiella pneumoniae liver abscess (KPLA) [4, 5].

Notably, KPLA is more susceptible to dissemination and metastatic infections than other bacterial liver abscesses (20.3% vs. 1.4%) [6], primarily attributed to hvKP infection [7]. The invasive liver abscess syndrome is defined as liver abscess combined with extrahepatic metastatic infections, such as endophthalmitis, meningitis, septic pulmonary embolism, osteomyelitis, and necrotizing fasciitis. Unfortunately, it is emerging as a globally devastating disease [8], which is difficult to treat and usually has a poor prognosis [9]. Endophthalmitis, the most common and serious complication, can cause severe vision impairment even with aggressive antibiotic therapy [10]. Additionally, metastatic infections may increase the frequency and length of intensive care unit (ICU) stays and in-hospital mortality [2].

Early identification of the pathogen is critical for treating liver abscesses, particularly in patients with severe systemic infections. However, blood or pus cultures usually take three to five days, potentially delaying treatment. Computed tomography (CT) is an effective method of diagnosing PLA and detecting its possible causes. Previous research attempted to differentiate between KPLA and NKPLA using traditional CT imaging features of liver abscesses, but they were mainly descriptive and required further clinical validation [6, 11, 12]. The radiologists’ experience and subjective assessment play a major role in determining the usefulness of CT-based screening. Radiomics is an emerging image analysis technology that can extract a large number of quantitative features from medical images and convert them into mineable data for clinical decision support [13]. To our knowledge, no research has created radiomics-based models for this purpose. To support the early diagnosis of KPLA and provide the groundwork for reasonable clinical decision-making in patients with PLA, this study endeavors to create an efficient and comprehensive nomogram that integrates clinical variables and radiomics features.

## Materials and methods

### Patients

**Figure 1.**
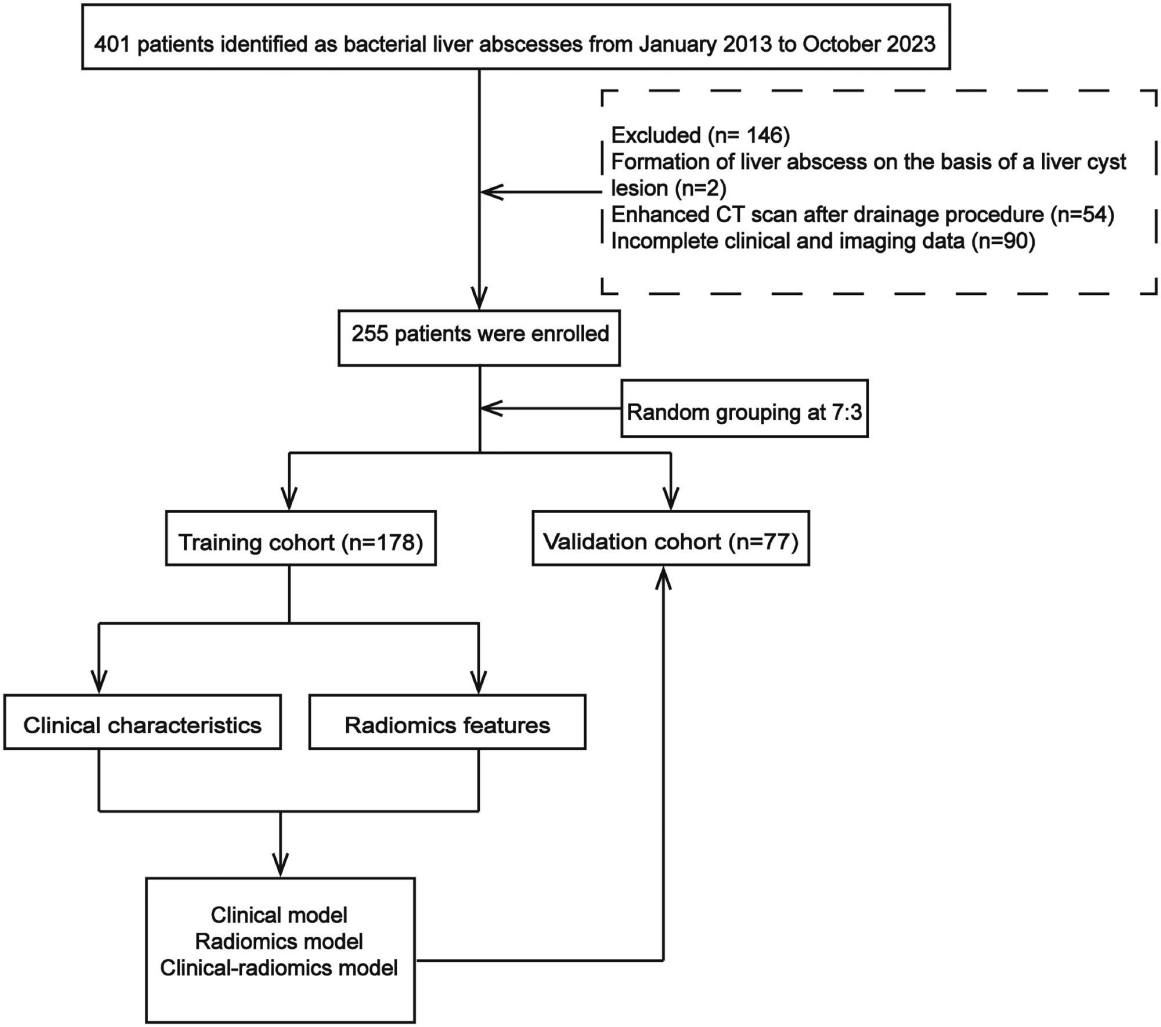
Flowchart of study patients.

This retrospective study was conducted following the Helsinki Declaration and approved by the Ethics Committee of Tongji Hospital (Approval ID: TJ-IRB20231234), which waived the informed consent requirement because of the retrospective and anonymous nature of the study. Patients with PLA diagnosed by CT images and culture results between 2013 and 2023 were included in this study. The following was a list of the criteria for inclusion: (1) Presence of one or more infectious lesions on images (such as ultrasound, CT, or magnetic resonance imaging (MRI)); (2) Positive pus or blood cultures revealing bacterial infection; (3) Enhanced CT images of the abdomen or liver; (4) Age ≥ 18 years old. Exclusion criteria were listed as follows: (1) Formation of liver abscess based on a liver tumor or liver cyst lesion (2) Enhanced CT scan following the drainage procedure; (3) Incomplete clinical or imaging data. Accordingly, 255 eligible patients with PLA were enrolled and randomly divided into the training (*n* = 178) and validation (*n* = 77) cohorts at a 7:3 ratio (Figure 1). Based on culture results, all patients were divided into two groups: the KPLA group (monomicrobial KP infection) and non-KPLA (NKPLA) group (monomicrobial non-KP infection or polymicrobial infection including KP infection).

### Clinical data collection

The following data were collected retrospectively: (1) patient demographics, including age and gender; (2) underlying diseases, including diabetes, liver malignancy, viral hepatitis, liver cirrhosis, and fatty liver, were assessed based on the patient’s history and laboratory tests; (3) clinical presentation, including fever and abdominal pain; (4) admission laboratory data, including white blood cell (WBC) count, neutrophil (NEUT) count, neutrophil percentage (NEUT%), red blood cell (RBC) count, hemoglobin (Hb) level, platelet (PLT) count, and the levels of alanine aminotransferase (ALT), aspartate aminotransferase (AST), alkaline phosphatase (ALP), glutamyl transpeptidase (GGT), direct bilirubin (DBIL), indirect bilirubin (IBIL), and C-reactive protein (CRP); (5) splenomegaly identified by CT, defined as exceeding the upper limit of the normal adult splenic length of 12 cm, width of 6 cm, and thicknesses of 4 cm; (6) the origin of infection: cryptogenic liver abscess. The infectious origin of liver abscesses was classified into five categories: biliary tract, portal vein, direct, hepatic artery, and cryptogenic. Cryptogenic liver abscess is usually identified after a full investigation has ruled out liver abscess that four obvious sources of infection may cause. The following lists of conditions were excluded from cryptogenic liver abscess: intra-abdominal infections (such as acute purulent appendicitis, as well as secondary to acute severe pancreatitis and biliary or intestinal fistula), recent abdominal surgery including invasive hepatobiliary operations within three months, underlying biliary disorders (such as cholecystitis, cholangitis, biliary stones, biliary tumors, and some conditions that cause biliary obstruction or stricture), obvious infections elsewhere before liver abscess formation, and liver trauma or injury, such as fishbone perforation.

### Image segmentation and radiomics feature extraction

Radiomics features were extracted from CT images of the liver in the arterial phase for this investigation. The CT acquisition parameters are described in Supplementary Material (1). Using ITK-SNAP software (version 3.8.0), a radiologist with more than fifteen years of experience in CT interpretation manually delineated the volume of interest (VOI) for each patient. If a CT scan revealed multiple abscess lesions in the liver, the one with the largest diameter was chosen. To reduce the heterogenic result from different scanning parameters, all images were resampled at the same voxel size of 1 × 1 × 1 mm^3^, and the grey level was simultaneously normalized before extraction. Supplementary Material (2) contains an exhaustive description of the extracted features (Table S1).

### Feature selections and radiomics score construction

All extracted features of each patient were normalized by z-score before the following analysis. One month later, thirty patients were randomly selected, and two radiologists repeated the same delineation procedure to assess the stability of the features using intra/interclass correlation coefficients (ICC). The features with ICC > 0.75 were considered strongly reproducible and dependable and included in the following study. The most valuable features were selected by the independent sample t-test and the least absolute shrinkage and selection operator (LASSO) regression in the training cohort. The optimal parameter was determined by 10-fold cross-validation. The selected features were used to construct a radiomics score (Rad-score), calculated for each patient *via* the linear combination of selected features weighted by their respective coefficients.

### Model construction and assessment

Univariate and multivariable logistic regression analyses were performed to determine independent clinical variables. Next, clinical, radiomics, and clinical-radiomics models were established based on the Rad-score and independent clinical predictors. The combined model is visualized as a nomogram *via* multivariate logistic regression. The prediction accuracy of the three models was evaluated *via* Harrell’s concordance index (C-index) and the area under the curve (AUC) of the receiver operating characteristic (ROC). The Delong test was conducted to compare the differences in the ROCs of the three models. The Hosmer-Lemeshow test and a calibration curve were used to evaluate the agreement between the predicted risks and the observed outcomes. Decision curve analysis (DCA) assessed the models’ net benefit and clinical utility.

### Statistical analysis

IBM SPSS Statistics (version 25), R software (version 4.3.1), and Python (version 3.9.6) were used for statistical analysis. The independent sample t-test or Mann-Whitney *U* test was performed for continuous variables, and the Chi-square test or Fisher’s exact test was used for categorical variables. Quantitative data was expressed as mean ± standard deviation for normal distribution, median (interquartile range) for non-normal distribution, and percentage for count data. Logistic regression analysis, nomogram, and calibration plots were executed with the ‘rms’ package. DCA was performed using the ‘rmda’ package. A two-sided P value < 0.05 was considered statistically significant.

## Results

### Clinical characteristics

The culture results revealed 106 patients with KPLA, 72 with NKPLA in the training cohort, 54 with KPLA and 23 with NKPLA in the validation cohort. There were 34 patients with monomicrobial infection and 61 with polymicrobial infection in the NKPLA group, where Escherichia coli and Enterococcus spp. are the most common (Table S2). Non-cryptogenic liver abscesses (*n* = 116) were mainly caused by biliary infections and portal vein infections in the study, with one or more of the following conditions: recent abdominal surgeries including invasive hepatobiliary operations (*n* = 42), underlying biliary diseases (*n* = 88), and some intra-abdominal infections (*n* = 7) (Tables S3 and S4). Patients with biliary disease or recent abdominal surgery were more common in the KPLA group than in the NKPLA group (*p* < 0.05) (Table S5). The baseline clinical characteristics of the patients with PLA in the training and validation cohorts are shown in Table 1. Except for viral hepatitis, most clinical characteristics did not differ significantly between the training and validation cohorts, indicating the comparability of the two patient cohorts.

**Table 1.** The clinical characteristics in the training cohort and validation cohort.

Variables	Training cohort	Validation cohort	P value
Age (years)	53.25 ± 1.12	57.49 ± 1.33	0.15
Sex, (n%)			
Male	130 (73.0%)	56 (72.7%)	0.96
Female	48 (27.0%)	21 (27.3%)	
Diabetes mellitus	66 (37.1%)	29 (37.7%)	0.93
Liver malignancy	25 (14.0%)	8 (10.4%)	0.43
Viral hepatitis	8 (4.5%)	13 (16.9%)	0.001*
Liver cirrhosis	12 (6.7%)	5 (6.5%)	0.94
Fatty liver	33 (18.3%)	12 (15.6%)	0.57
Fever	166 (93.3%)	74 (96.1%)	0.55
Abdominal pain	54 (30.3%)	30 (39.0%)	0.18
Splenomegaly	40 (22.5%)	14 (18.2%)	0.44
Cryptogenic liver abscess	92 (51.7%)	47 (61.0%)	0.17
WBC count (×10^9/L)	12.33 (8.92, 17.29)	13.44 (9.74, 17.51)	0.60
NEUT count (×10^9/L)	10.04 (7.51, 15.50)	11.01 (7.82, 15.05)	0.46
NEUT% (%)	85.25 (78.20, 90.43)	85.00 (79.70, 92.05)	0.26
RBC count (×10^12/L)	3.81 ± 0.06	3.85 ± 0.08	0.65
Hb (g/L)	111.94 ± 1.79	113.13 ± 2.49	0.71
PTL count (×10^9/L)	183.50 (110.75, 294.00)	174.00 (88.50, 296.00)	0.67
ALT (U/L)	39.5 (24.00, 98.00)	56.00 (24.00, 84.00)	0.74
AST (U/L)	41.00 (22.75, 75.00)	45.00 (22.50, 83.50)	0.70
ALP (U/L)	171.50 (118.00,252,75)	186.00 (115.50, 275.00)	0.55
GGT (U/L)	109.00 (62.00, 193.75)	136.00 (65.00, 223.50)	0.75
DBIL (μmol/L)	9.15 (5.50, 10.73)	9.40 (5.35,20.25)	0.74
IBIL (μmol/L)	5.70 (3.90, 9.50)	5.70 (3.15, 8.95)	0.41
CRP (mg/L)	156.40 (104.85, 225.58)	148.00 (102.10, 218.85)	0.60

*p < 0.05, statistically significant results.

ALT: alanine aminotransferase; AST: aspartate aminotransferase; ALP: alkaline phosphatase; CRP: C-reactive protein; DBIL: direct bilirubin; GGT: glutamyl transpeptidase; Hb: hemoglobin; IBIL: indirect bilirubin; NEUT: neutrophil; NEUT%: neutrophil percentage; PLT: platelet; RBC: red blood cell; WBC: white blood cell.

### Radiomics feature selection and Rad-score construction

**Figure 2.**
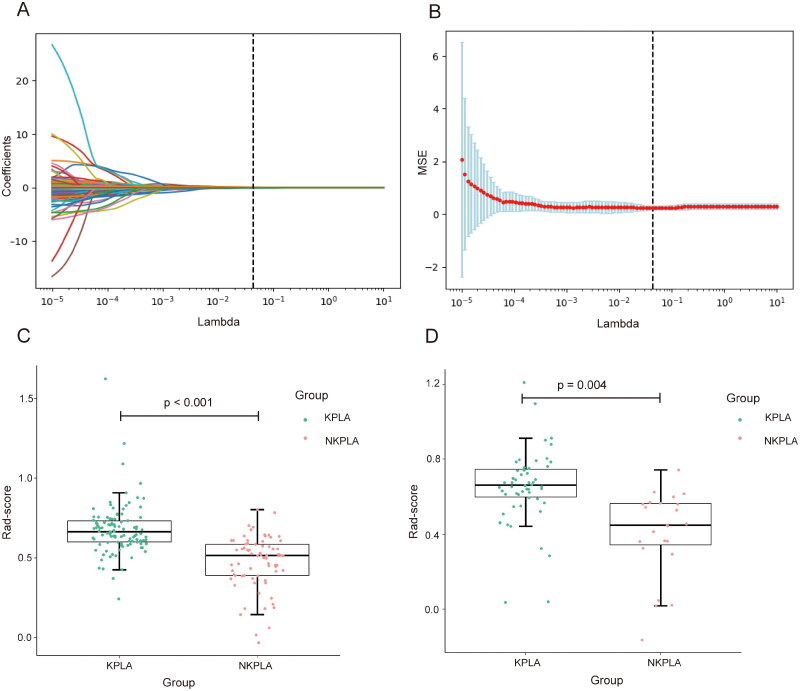
Radiomics feature selection and Rad-score comparison. (A) Selection of tuning parameter log (λ) selection in the LASSO model by 10-fold cross-validation according to minimum criteria. The optimal λ value of 0.043 was selected. (B) LASSO coefficients profiles of the radiomics features. Seven features with non-zero coefficients are shown in the plot. (C,D) Comparison of Rad-score between the KPLA and NKPLA groups. There was a significant difference in the Rad-score between the KPLA and NKPLA groups in the training (p < 0.001) and validation (p = 0.004) cohorts. LASSO: least absolute shrinkage and selection operator; KPLA: Klebsiella pneumoniae liver abscess; NKPLA: non-Klebsiella pneumoniae liver abscess.

A total of 851 radiomics features were extracted from enhanced CT images of liver abscesses. Firstly, 265 features with ICCs less than 0.75 were excluded, and the remaining 586 features were highly reproducible and robust. Next, 103 features were filtered by the independent sample t-test, and seven optimal radiomics features were selected by LASSO regression (Figure 2A,B). Finally, as detailed in Supplementary Material (4), the Rad-score was calculated. The Rad-score differed significantly between the KPLA and NKPLA groups, with *p* < 0.001 and *p* = 0.004 in the training and validation cohorts, respectively (Figure 2C,D). Patients with KPLA tended to have higher Rad-score.

### Model construction and assessment

**Figure 3.**
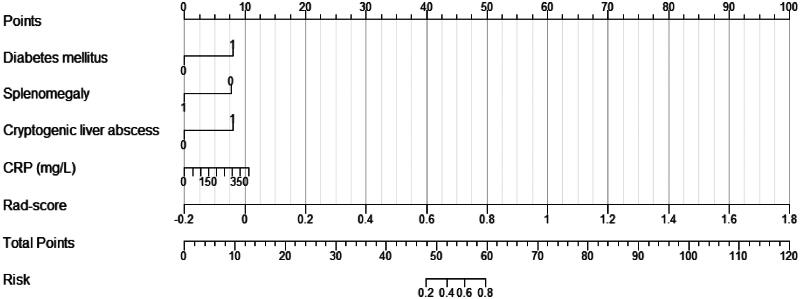
Development of the nomogram for predicting the probability of KPLA.

Univariate and multivariate logistic regression analyses are shown in Table 2. Univariate analysis screened out some clinical factors significantly associated with KPLA, including younger males, the presence of diabetes mellitus and fatty liver, absence of liver malignancy, liver cirrhosis and splenomegaly, the presence of cryptogenic liver abscess, higher RBC count and Hb and CRP levels, and lower ALP, DBIL, and IBIL levels (all *p* < 0.05). These variables were included in the multivariate logistic analysis, and it showed that the presence of diabetes mellitus and cryptogenic liver abscess, absence of splenomegaly, and CRP level were independent predictors for KPLA (all *p* < 0.05). The higher the level of CRP in the blood, the greater the likelihood of KPLA. Consequently, the clinical-radiomics nomogram was established using multivariate logistic regression (Figure 3), incorporating diabetes mellitus, splenomegaly, cryptogenic liver abscess, CRP level, and a Rad-score.

**Table 2.** Univariate and multivariate analyses of clinical variables in the training cohort.

	Univariate logistic regression		Multivariate logistic regression	
Variables	OR (95%CI)	P value	OR (95%CI)	P value
Age (years)	0.97 (0.95, 0.99)	0.004*		
Male, (n%)	2.43 (1.24, 4.77)	0.01*		
Diabetes mellitus	4.54 (2.23, 9.24)	<0.001*	6.33 (2.33-17.22)	<0.001*
Liver malignancy	0.10 (0.03, 0.29)	<0.001*		
Viral hepatitis	1.14 (0.26, 4.92)	0.86		
Liver cirrhosis	0.20 (0.05, 0.78)	0.02*		
Fatty liver	2.47 (1.04, 5.84)	0.04*		
Fever	0.47 (.0.12, 1.79)	0.27		
Abdominal pain	0.98 (0.51, 1.88)	0.96		
Splenomegaly	0.27 (0.13, 0.56)	0.001*	0.23 (0.08-.069)	0.008*
Cryptogenic liver abscess	10.09 (4.95, 20.55)	<0.001*	4.60 (1.84-11.49)	0.001*
WBC count (×10^9/L)	0.97 (0.93, 1.02)	0.25		
NEUT count (×10^9/L)	0.99 (0.96, 1.03)	0.56		
NEUT% (%)	0.97 (0.94, 1.01)	0.10		
RBC count (×10^12/L)	2.94 (1.83, 4.71)	<0.001*		
Hb (g/L)	1.04 (1.02, 1.05)	<0.001*		
PTL count, (×10^9/L)	1.00 (0.10, 1.00)	0.13		
ALT (U/L)	1.004 (1.000, 1.008)	0.05		
AST (U/L)	1.00 (0.99, 1.01)	0.23		
ALP (U/L)	0.997 (0.994, 0.999)	0.01*		
GGT (U/L)	0.998 (0.995, 1.000)	0.12		
DBIL (μmol/L)	0.994 (0.987, 1.000)	0.047*		
IBIL (μmol/L)	0.96 (0.92, 1.00)	0.04*		
CRP (mg/L)	1.007 (1.003, 1.012)	<0.001*	1.008 (1.002-1.013)	0.007*

*p < 0.05, statistically significant results from logistic regression analysis.

**Figure 4.**
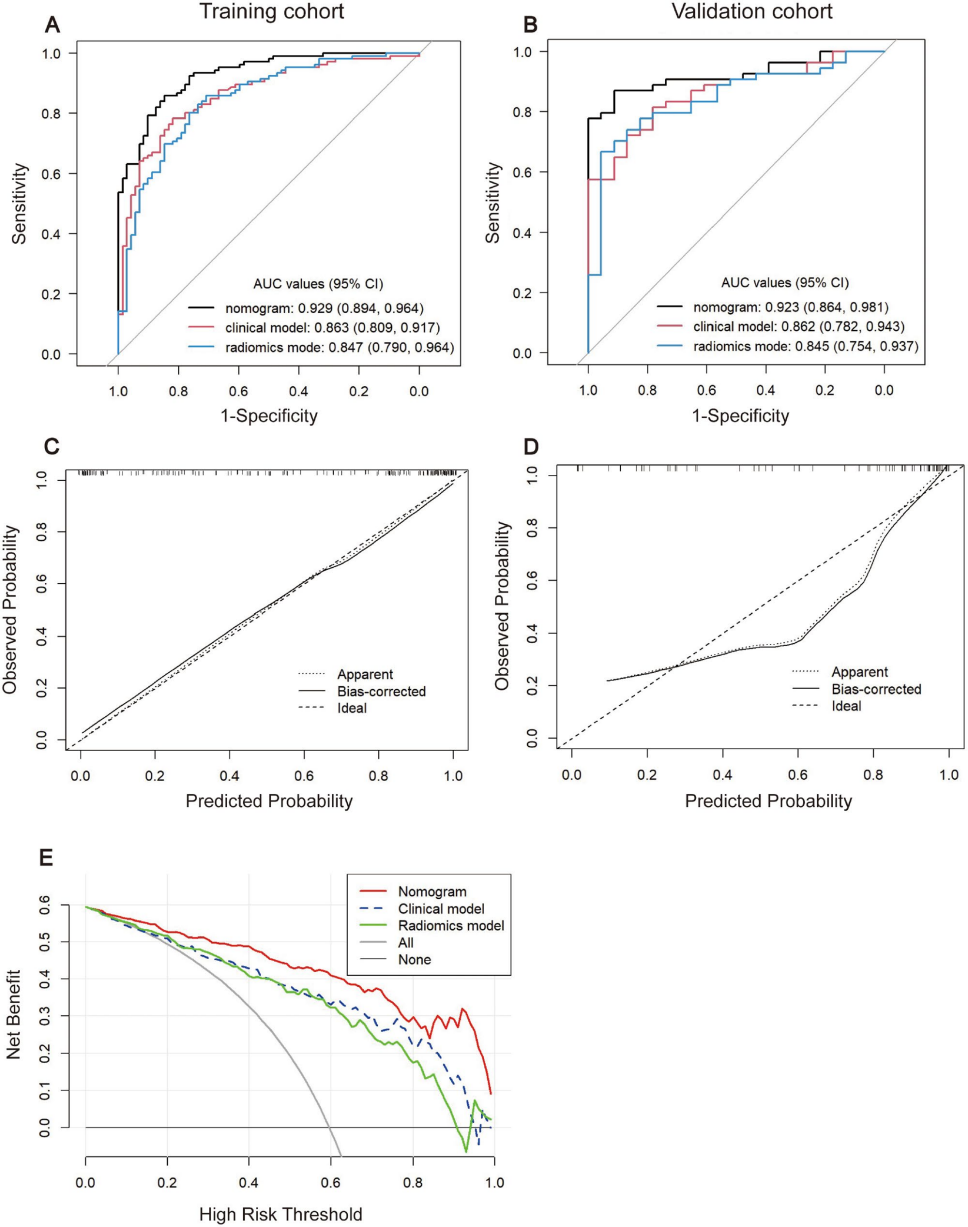
Predictive performance of the three models. (A,B) ROC curves of three models in the training and validation cohorts. (C,D) Calibration curves of the nomogram in the training and validation cohorts. (E) Decision curve analysis for three models in the training cohort. ROC: receiver operating characteristic curve.

Figure 4(A,B) showed the predictive abilities of three models in two cohorts. The nomogram yielded an AUC of 0.929 (95% CI, 0.894-0.964) and 0.923 (95% CI, 0.864-0.981) in the training and validation cohorts, respectively. Similarly, the C-index of the nomogram was 0.918 and 0.921 in the training and validation cohorts, respectively (Table S6). Furthermore, the nomogram had better predictive performance than the clinical model or radiomics model in the training (AUC: 0.929 vs. 0.863 or 0.847, *p* = 0.001 and 0.001, respectively) and validation (AUC: 0.923 vs. 0.862 or 0.845, *p* = 0.16 and 0.01, respectively) cohorts. In contrast, there was no significant difference in AUC between the clinical and radiomics models in the training and validation cohorts (*p* = 0.70 and 0.80, respectively).

The calibration curves (Figure 4C,D) showed good consistency between the nomogram-predicted probabilities and the actual probabilities, with the Hosmer-Lemeshow test of *p* = 0.54 and *p* = 0.15 in the training and validation cohorts, respectively, showing good consistency. The decision curve analysis (Figure 4E) proved that when the patients’ threshold probability is more than 5.5%, applying the nomogram would result in a net benefit surpassing either the treat-all or treat-none method. Within this range, the nomogram always showed a greater net benefit than the clinical and radiomics models, indicating that the nomogram had superior clinical utility.

## Discussion

Klebsiella pneumoniae liver abscess spreads worldwide, with a high risk of complicated metastatic infections [8, 14]. Zhang et al. [4] reported that approximately half of KPLA occurred in healthy individuals, indicating a strong association with hvKP and the possibility of invasive lethal liver abscesses. Early detection of KPLA is critical for treating and evaluating complications, as they progress quickly and have a poor prognosis. This study developed a noninvasive nomogram by retrospectively analyzing the differences between KPLA and NKPLA in clinical and radiomics features to aid in the early diagnosis of KPLA.

Prior studies have reported some conventional CT features associated with KPLA, such as single, thin-walled, multiloculated abscesses, solid appearance, internal necrotic debris, absence of rim enhancement, and thrombophlebitis [6, 11, 12, 15]. Although conventional CT features were not included in this study, seven radiomic features were chosen to generate a Rad-score with good predictive power for KPLA. Compared to conventional imaging features, radiomics can detect invisible disease features by extracting innumerable quantitative features from tomographic images [13]. However, radiomics is not a panacea for clinical decision-making. To enhance diagnostic performance, a nomogram was developed by combining radiomics features and clinical factors, which included five comprehensive and easily accessible variables: the Rad-score, diabetes mellitus, cryptogenic liver abscess, CRP level, and splenomegaly.

Our study showed that KPLA was strongly associated with cryptogenic liver abscesses and was rarely accompanied by biliary disease and recent surgical history, consistent with previous studies [2, 6, 16]. Lee et al. [12] found that the absence of underlying biliary disease, metastatic infection, and two conventional CT features of thin-walled abscess and absence of rim enhancement were the most significant predictors of KPLA, with at least three of these signs achieving a high specificity of 96.5% but a poor sensitivity of 39.1%. KPLA of unknown origins may be related to KP’s virulence and pathogenic characteristics. Intestinal colonization of KP in humans appears to be a key contributing factor to primary liver abscess, which allows hvKP to cross the intestinal barrier under some circumstances and subsequently invade the liver and be sequestered by Kuffer cells [17–19]. Capsular polysaccharide (K antigen), a major virulence determinant of hvKP, makes it resistant to phagocytosis and intracellular killing by phagocytes, thus susceptible to dissemination and the formation of cryptogenic liver abscesses caused by KP [20–22].

Chen et al. [16] showed that Klebsiella pneumoniae was the most common pathogen in patients with cryptogenic liver abscesses or diabetic patients with liver abscesses. There were contradictory findings from several studies about the relationship between KPLA and diabetes mellitus [2, 6, 23, 24]. This study demonstrated that diabetes mellitus is an independent risk factor for KPLA and that diabetic patients with liver abscesses are more likely to be exposed to monomicrobial KP infections. Poor glycemic control in diabetic patients selectively impairs neutrophil phagocytosis of capsular serotype K1/K2 KP, thus potentially increasing the risk of KPLA formation and metastatic infections [25, 26].

Markedly elevated CRP levels are often found in patients with KPLA [27, 28], but the link between the two has not been demonstrated in prior studies. Our study demonstrated that the higher the CRP level, the higher the likelihood of liver abscesses infected by KP. This may be due to the K1 and K2 strains’ resistance to intracellular killing by neutrophils and in serum, which contributes to dissemination and more pronounced inflammation [21, 22]. CT is considered a reliable and accurate method for assessing the splenic volume [29]. This study found that 21.2% of patients with PLA had splenomegaly. Interestingly, the absence of splenomegaly was shown to be important in predicting KPLA despite not being mentioned in previous studies. The result may be associated with a significantly higher percentage of chronic liver diseases in patients with NKPLA, such as liver malignancy and cirrhosis.

Blood or pus culture is the standard method for identifying pathogens and guiding antibiotic administration. Nonetheless, obtaining results usually takes several days, and the positive rate is not ideal [30]. Sometimes, liver abscesses can present with a multicompartmental or solid appearance, especially in KPLA [11], making them difficult for drainage and pus cultures. However, delays in pathogen identification probably lead to untimely and inappropriate antimicrobial therapy and ultimately to severe complications, even death. As expected, the nomogram contributed to the early diagnosis of KPLA with excellent predictive performance. KP is currently sensitive to most antimicrobial agents but is intrinsically resistant to ampicillin [31]. Still, some hypervirulent isolates with resistance are increasingly being detected, which can pose a great threat to public health, thus necessitating the rational use of antibiotics [32]. Hopefully, the proposed nomogram will guide appropriate empirical antibiotic therapy for PLA pending culture results.

Our study had some limitations. First, this study was conducted retrospectively based on small-scale data from a single center. Thus, multicenter, prospective, and larger-scale studies are required to confirm the nomogram’s feasibility and reliability. Second, to achieve higher diagnostic accuracy of KPLA, various models will be developed based on deep learning methods and other image imaging modalities like ultrasound and MRI.

## Conclusions

We were the first to develop and validate a clinical-radiomics nomogram with promising diagnostic potential for the prediction of KPLA. It is hoped that early application of the nomogram could facilitate clinical decision-making in patients with PLA to some extent.

## Supplementary Material

Clean copy - Supplementary_Material (1).docx

## Data Availability

The data generated or analyzed during this study are available from the corresponding author upon reasonable request.

## Abbreviations

AUC: the area under the curve
ALT: alanine aminotransferase
AST: aspartate aminotransferase
ALP: alkaline phosphatase
cKP: classic Klebsiella pneumoniae
CT: computed tomography
CRP: C-reactive protein
C-index: Harrell’s concordance index
DBIL: direct bilirubin
DCA: decision curve analysis
GGT: glutamyl transpeptidase
hvKP: hypervirulent Klebsiella pneumoniae
Hb: hemoglobin
IBIL: indirect bilirubin
ICU: intensive care unit
ICC: intra-/inter-class correlation coefficient
KP: Klebsiella pneumoniae
KPLA: Klebsiella pneumoniae liver abscess
PLA: pyogenic liver abscess
LASSO: least absolute shrinkage and selection operator
MRI: magnetic resonance imaging
NKPLA: non-Klebsiella pneumoniae liver abscess
NEUT: neutrophil
NEUT%: neutrophil percentage
PLT: platelet
ROC: receiver operating characteristic curve
Rad-score: radiomics score
RBC: red blood cell
VOI: the volume of interest
WBC: white blood cell

## References

[1] Wang JH, Liu YC, Lee SS, et al. Primary liver abscess due to Klebsiella pneumoniae in Taiwan. Clin Infect Dis. 1998;26(6):1434–1438. doi: 10.1086/516369. 9636876

[2] Qian Y, Wong CC, Lai S, et al. A retrospective study of pyogenic liver abscess focusing on Klebsiella pneumoniae as a primary pathogen in China from 1994 to 2015. Sci Rep. 2016;6(1):38587. doi: 10.1038/srep38587. 27929082 PMC5144064

[3] Choby JE, Howard-Anderson J, Weiss DS. Hypervirulent Klebsiella pneumoniae: clinical and molecular perspectives. J Intern Med. 2020;287(3):283–300. doi: 10.1111/joim.13007. 31677303 PMC7057273

[4] Zhang S, Zhang X, Wu Q, et al. Clinical, microbiological, and molecular epidemiological characteristics of Klebsiella pneumoniae-induced pyogenic liver abscess in southeastern China. Antimicrob Resist Infect Control. 2019;8(1):166. doi: 10.1186/s13756-019-0615-2. 31673355 PMC6819602

[5] Fung C-P, Chang F-Y, Lee S-C, et al. A global emerging disease of Klebsiella pneumoniae liver abscess: is serotype K1 an important factor for complicated endophthalmitis? Gut. 2002;50(3):420–424. doi: 10.1136/gut.50.3.420. 11839725 PMC1773126

[6] Lee NK, Kim S, Lee JW, et al. CT differentiation of pyogenic liver abscesses caused by Klebsiella pneumoniae vs. non-Klebsiella pneumoniae. BJR. 2011;84(1002):518–525. doi: 10.1259/bjr/23004588. 21081584 PMC3473636

[7] Shon AS, Bajwa RPS, Russo TA. Hypervirulent (hypermucoviscous) Klebsiella pneumoniae: a new and dangerous breed. Virulence. 2013;4(2):107–118. doi: 10.4161/viru.22718. 23302790 PMC3654609

[8] Siu LK, Yeh K-M, Lin J-C, et al. Klebsiella pneumoniae liver abscess: a new invasive syndrome. Lancet Infect Dis. 2012;12(11):881–887. doi: 10.1016/S1473-3099(12)70205-0. 23099082

[9] Lin Y-T, Liu C-J, Chen T-J, et al. Long-term mortality of patients with septic ocular or central nervous system complications from pyogenic liver abscess: a population-based study. PLoS One. 2012;7(3):e33978. doi: 10.1371/journal.pone.0033978. 22479491 PMC3313956

[10] Yang C-S, Tsai H-Y, Sung C-S, et al. Endogenous Klebsiella endophthalmitis associated with pyogenic liver abscess. Ophthalmology. 2007;114(5):876–880. doi: 10.1016/j.ophtha.2006.12.035. 17467526

[11] Alsaif HS, Venkatesh SK, Chan DSG, et al. CT appearance of pyogenic liver abscesses caused by Klebsiella pneumoniae. Radiology. 2011;260(1):129–138. doi: 10.1148/radiol.11101876. 21460028

[12] Lee JH, Jang YR, Ahn SJ, et al. A retrospective study of pyogenic liver abscess caused primarily by Klebsiella pneumoniae vs. non-Klebsiella pneumoniae: CT and clinical differentiation. Abdom Radiol. 2020;45(9):2669–2679. doi: 10.1007/s00261-019-02389-2. 31894381

[13] Gillies RJ, Kinahan PE, Hricak H. Radiomics: images are more than pictures; they are data. Radiology. 2016;278(2):563–577. doi: 10.1148/radiol.2015151169. 26579733 PMC4734157

[14] Moore R, O’Shea D, Geoghegan T, et al. Community-acquired Klebsiella pneumoniae liver abscess: an emerging infection in Ireland and Europe. Infection. 2013;41(3):681–686. doi: 10.1007/s15010-013-0408-0. 23381876

[15] Hui JYH, Yang MKW, Cho DHY, et al. Pyogenic liver abscesses caused by Klebsiella pneumoniae: US appearance and aspiration findings. Radiology. 2007;242(3):769–776. doi: 10.1148/radiol.2423051344. 17325065

[16] Chen S-C, Wu W-Y, Yeh C-H, et al. Comparison of Escherichia coli and Klebsiella pneumoniae liver abscesses. Am J Med Sci. 2007;334(2):97–105. doi: 10.1097/MAJ.0b013e31812f59c7. 17700198

[17] Fung C-P, Lin Y-T, Lin J-C, et al. Klebsiella pneumoniae in the gastrointestinal tract and pyogenic liver abscess. Emerg Infect Dis. 2012;18(8):1322–1325. doi: 10.3201/eid1808.111053. 22840473 PMC3414011

[18] Tu Y-C, Lu M-C, Chiang M-K, et al. Genetic requirements for Klebsiella pneumoniae-induced liver abscess in an oral infection model. Infect Immun. 2009;77(7):2657–2671. doi: 10.1128/IAI.01523-08. 19433545 PMC2708586

[19] Wanford JJ, Hames RG, Carreno D, et al. Interaction of Klebsiella pneumoniae with tissue macrophages in a mouse infection model and ex-vivo pig organ perfusions: an exploratory investigation. Lancet Microbe. 2021;2(12):e695–e703. doi: 10.1016/S2666-5247(21)00195-6. 34901898 PMC8641047

[20] Yeh K-M, Kurup A, Siu LK, et al. Capsular serotype K1 or K2, rather than magA and rmpA, is a major virulence determinant for Klebsiella pneumoniae liver abscess in Singapore and Taiwan. J Clin Microbiol. 2007;45(2):466–471. doi: 10.1128/JCM.01150-06. 17151209 PMC1829066

[21] Fung C-P, Chang F-Y, Lin J-C, et al. Immune response and pathophysiological features of Klebsiella pneumoniae liver abscesses in an animal model. Lab Invest. 2011;91(7):1029–1039. doi: 10.1038/labinvest.2011.52. 21464821

[22] Lin J-C, Chang F-Y, Fung C-P, et al. Do neutrophils play a role in establishing liver abscesses and distant metastases caused by Klebsiella pneumoniae? PLoS One. 2010;5(11):e15005. doi: 10.1371/journal.pone.0015005. 21151499 PMC2994827

[23] Liu J, Liu Y, Li C, et al. Characteristics of Klebsiella pneumoniae pyogenic liver abscess from 2010-2021 in a tertiary teaching hospital in South China. J Glob Antimicrob Resist. 2024;36:210–216. doi: 10.1016/j.jgar.2023.12.024. 38154752

[24] Kim JK, Chung DR, Wie SH, et al. Risk factor analysis of invasive liver abscess caused by the K1 serotype Klebsiella pneumoniae. Eur J Clin Microbiol Infect Dis. 2009;28(1):109–111. doi: 10.1007/s10096-008-0595-2. 18663497

[25] Lin J-C, Siu LK, Fung C-P, et al. Impaired phagocytosis of capsular serotypes K1 or K2 Klebsiella pneumoniae in type 2 diabetes mellitus patients with poor glycemic control. J Clin Endocrinol Metab. 2006;91(8):3084–3087. doi: 10.1210/jc.2005-2749. 16720670

[26] Wang H, Guo Y, Yan B, et al. Development and validation of a prediction model based on clinical and CT features for invasiveness of K. pneumoniae liver abscess. Eur Radiol. 2022;32(9):6397–6406. doi: 10.1007/s00330-022-08740-4. 35364715

[27] Yang F, Wang L, Zhao Q, et al. Epidemiological features of Klebsiella pneumoniae infection in the hepatobiliary system of patients in Yantai, China, based on clinical and genetic analyses. Infect Drug Resist. 2022;15:3427–3436. doi: 10.2147/IDR.S369988. 35800122 PMC9253619

[28] Lee SS-J, Chen Y-S, Tsai H-C, et al. Predictors of septic metastatic infection and mortality among patients with Klebsiella pneumoniae liver abscess. Clin Infect Dis. 2008;47(5):642–650. doi: 10.1086/590932. 18643760

[29] Yetter EM, Acosta KB, Olson MC, et al. Estimating splenic volume: sonographic measurements correlated with helical CT determination. AJR Am J Roentgenol. 2003;181(6):1615–1620. doi: 10.2214/ajr.181.6.1811615. 14627584

[30] Chemaly RF, Hall GS, Keys TF, et al. Microbiology of liver abscesses and the predictive value of abscess gram stain and associated blood cultures. Diagn Microbiol Infect Dis. 2003;46(4):245–248. doi: 10.1016/s0732-8893(03)00088-9. 12944014

[31] Ren Y, Wang H, Chang Z, et al. Clinical and computed tomography features of extended-spectrum β-lactamase-producing Klebsiella pneumoniae liver abscess. BMC Infect Dis. 2020;20(1):416. doi: 10.1186/s12879-020-05142-z. 32539687 PMC7296744

[32] Lee C-R, Lee JH, Park KS, et al. Antimicrobial Resistance of Hypervirulent Klebsiella pneumoniae: epidemiology, Hypervirulence-Associated Determinants, and Resistance Mechanisms. Front Cell Infect Microbiol. 2017;7:483. doi: 10.3389/fcimb.2017.00483. 29209595 PMC5702448

