# Supplementary material for: Development and validation of a clinical-radiomics nomogram for the early prediction of Klebsiella pneumoniae liver abscess
Source: Ann Med. 2024 Oct 11;56(1):2413923. doi: 10.1080/07853890.2024.2413923 (PMC11485847; doi:10.1080/07853890.2024.2413923)
Supplement: Clean copy - Supplementary_Material (1).docx [file IANN_A_2413923_SM6945.docx]

**Supplementary Material (1). CT acquisition parameters:**

All the patients were scanned using a 64-, 256- or 320-multidetector CT scanner (SOMATOM Force, Siemens, Germany; Revolution, GE Healthcare, USA; Aquilion ONE, Canon Medical Systems, Japan), with similar scan parameters. The scan parameters were as follows: tube voltage, 100-120 kv; tube current, auto; rotation time, 0.5-0.8 seconds; pitch, 0.6-0.9; matrix, 512×512; slice thickness, 1.25mm. All patients received an injection of ionic contrast material at a total dose of 30-60ml into the cubital vein at a rate of 2.5-3.5 mL/s, followed by flushing with 30 mL saline solution. The arterial phase CT scans were performed with a 20-second delay after contrast injection.

**Supplementary Material (2). The detailed introduction on extracted radiomics features.**

The radiomics features were extracted from the VOIs by Pyradiomics (an open-source Python package). The feature extraction methods in this study included original feature classes and wavelet filter classes. These original features included Shape, First-order statistics, and Texture, including Gray-Level Cooccurrence Matrix (GLCM), Gray-Level Run Length Matrix (GLRLM), Gray-Level Size Zone Matrix (GLSZM), Neighboring Gray Tone Difference Matrix (NGTDM), and Gray Level Dependence Matrix (GLDM). Wavelet filtering was applied to First-order statistics and Texture for all images. A total of 851 radiomics features were extracted, as detailed in **Table S1**.

|  | Original | Wavelet |
| --- | --- | --- |
| Shape (14) | 14×1 | - |
| First-order statistics (18) | 18×1 | 18×8 |
| Texture (75) | 75×1 | 75×8 |

**Supplementary Material (3).**

**Table S2.** Bacterial strains in the NKPLA group

| **Bacterial strain** | **Patients (n/95, %)** |
| --- | --- |
| Escherichia coli | 45 (47.37%) |
| Enterococcus spp. | 35 (36.84%) |
| Streptococcus spp. | 20 (21.05%) |
| Klebsiella pneumoniae | 11 (11.58%) |
| Pseudomonas aeruginosa | 10 (10.53%) |
| Other Enterobacteriaceae | 9 (9.47%) |
| Staphylococcus spp. | 8 (8.42%) |
| Acinetobacter baumannii | 3 (3.16%) |

**Table S3.** Distribution of cryptogenic liver abscesses in the study.

| **Group** | **Cryptogenic liver abscess (n, %)** | **Non-cryptogenic liver abscess (n, %)** |
| --- | --- | --- |
| KPLA | 118 (84.9%) | 42 (36.2%) |
| NKPLA | 21 (15.1%) | 74 (63.8%) |
| All | 139 | 116 |

**Table S4.** Possible etiologies of liver abscesses in patients with non-cryptogenic liver abscesses.

| **Etiologies** | **Patients (n)** |
| --- | --- |
| **Single etiology** |  |
| abdominal surgery | 19 |
| biliary disease | 62 |
| direct liver infection | 0 |
| intra-abdominal infection | 3 |
| ^#^obvious infection elsewhere | 0 |
| **Two or more etiologies** |  |
| abdominal surgery & biliary disease | 20 |
| abdominal surgery & direct liver infection | 1 |
| biliary disorder & direct liver infection | 2 |
| biliary disorder & intra-abdominal infection | 2 |
| abdominal surgery & biliary disease & intra-abdominal infection | 2 |
| **Total** |  |
| Total of abdominal surgery | 42 |
| Total of biliary disease | 88 |
| Total of direct liver infection | 3 |
| Total of intra-abdominal infection | 7 |

^#^obvious infection elsewhere, obvious infection outside the intra-abdominal cavity before liver abscess formation.

**Table S5.** The comparison of the KPLA and NKPLA groups regarding underlying biliary disease and recent surgical history in the study.

| **Variables** | **KPLA (n = 160)** | **NKPLA (n = 95)** | **p-value** |
| --- | --- | --- | --- |
| Biliary disease | 37 (23.1%) | 51 (53.7%) | < 0.001 |
| Abdominal surgery | 7 (4.4%) | 35 (36.8%) | < 0.001 |

**Table S6**. Predictive performance of three models in the training and validation cohorts.

|  | **Training cohort** | | | | |  | **Validation cohort** | | | |  |
| --- | --- | --- | --- | --- | --- | --- | --- | --- | --- | --- | --- |
| **Model** | C-index | AUC (95%CI) | Sensitivity | Specificity | |  | C-index | AUC (95%CI) | Sensitivity | Specificity | |
| **Nomogram** | 0.917 | 0.929  (0.894-0.964) | 0.858 | 0.847 |  | | 0.922 | 0.923  (0.864-0.981) | 0.870 | 0.913 | |
| **Clinical model** | 0.853 | 0.863  (0.809-0.917) | 0.783 | 0.819 |  | | 0.862 | 0.862  (0.782-0.943) | 0.815 | 0.783 | |
| **Radiomics model** | 0.847 | 0.847  (0.790-0.964) | 0.858 | 0.708 |  | | 0.847 | 0.845  (0.754-0.937) | 0.667 | 0.957 |  |

**Abbreviations:** C-index, Harrell’s concordance index; AUC, the area under the receiver operating characteristic curve.

**Supplementary Material (4). Radiomics score (Rad-score) calculation formula:**

Rad-score = 0.595506 + wavelet-LLH_glcm_ClusterProminence × 0.017344 + wavelet-LLH_glcm_Imc1 × -0.016904 + wavelet-LHL_firstorder_Uniformity × -0.000315 + wavelet-LHL_gldm_GrayLevelVariance × 0.006202 + wavelet-LHL_gldm_HighGrayLevelEmphasis × -0.080661 + wavelet-HLL_firstorder_Mean × -0.064262 + wavelet-LLL_firstorder_InterquartileRange × 0.109963.
